# TWIK-1/TASK-3 heterodimeric channels contribute to the neurotensin-mediated excitation of hippocampal dentate gyrus granule cells

**DOI:** 10.1038/s12276-018-0172-4

**Published:** 2018-11-12

**Authors:** Jae Hyouk Choi, Oleg Yarishkin, Eunju Kim, Yeonju Bae, Ajung Kim, Seung-Chan Kim, Kanghyun Ryoo, Chang-Hoon Cho, Eun Mi Hwang, Jae-Yong Park

**Affiliations:** 1 Korea Institute of Science and Technology (KIST), Center for Functional Connectomics, Seoul, 02792 Republic of Korea; 2 Division of Bio-Medical Science and Technology, KIST School, Korea University of Science and Technology, Seoul, 02792 Republic of Korea; 3 School of Biosystem and Biomedical Science, College of Health Science, Korea University, Seoul, 02841 Republic of Korea; 4 KHU-KIST Department of Converging Science and Technology, Kyung Hee University, Seoul, 02447 Republic of Korea

## Abstract

Two-pore domain K^+^ (K2P) channels have been shown to modulate neuronal excitability. The physiological role of TWIK-1, the first identified K2P channel, in neuronal cells is largely unknown, and we reported previously that TWIK-1 contributes to the intrinsic excitability of dentate gyrus granule cells (DGGCs) in mice. In the present study, we investigated the coexpression of TWIK-1 and TASK-3, another K2P member, in DGGCs. Immunohistochemical staining data showed that TASK-3 proteins were highly localized in the proximal dendrites and soma of DGGCs, and this localization is similar to the expression pattern of TWIK-1. TWIK-1 was shown to associate with TASK-3 in DGGCs of mouse hippocampus and when both genes were overexpressed in COS-7 cells. shRNA-mediated gene silencing demonstrated that TWIK-1/TASK-3 heterodimeric channels displayed outwardly rectifying currents and contributed to the intrinsic excitability of DGGCs. Neurotensin–neurotensin receptor 1 (NT–NTSR1) signaling triggered the depolarization of DGGCs by inhibiting TWIK-1/TASK-3 heterodimeric channels, causing facilitated excitation of DGGCs. Taken together, our study clearly showed that TWIK-1/TASK-3 heterodimeric channels contribute to the intrinsic excitability of DGGCs and that their activities are regulated by NT–NTSR1 signaling.

## Nerve cell communication: Controlling excitation

Inhibition of potassium channels by the hormone neurotensin (NT) increases the excitability of the dentate gyrus granule cells (DGGCs) of the hippocampus in the mouse brain. A study led by Jae-Yong Park at Korea University and Eun Mi Hwang at Korea Institute of Science and Technology, Seoul, found that the potassium channels TWIK-1 and TASK-3 associate with each other on the membrane of DGGCs. They showed that NT-mediated activation of NT receptor 1 regulates the electrical properties of DGGCs by inhibiting the TWIK-1/TASK-3 complex. This leads to a decrease in the number of potassium ions pumped out of DGGCs and increases in the DGGCs’ ability to transmit information. Further studies on the mechanisms regulating these channels will contribute to our understanding of how they influence information processing in the hippocampus.

## Introduction

Two-pore domain K^+^ (K2P) channels have long been recognized as essential for background K^+^ conductance in cells and control both neuronal resting membrane potential (RMP) and neuronal excitability^1^. The activity of these channels is modulated by a variety of physical and chemical factors, including temperature, membrane stretching, pH, protein kinases, polyunsaturated fatty acids, hormones, and neurotransmitters^1^. The K2P channel family has diverse functions in adrenal gland development, thermal and mechanical nociception, and sensitivity to volatile anesthetics^1^. The first identified K2P channel family member was TWIK-1 (Tandem of pore domains in Weak Inward rectifying K^+^ channel 1, often referred to as KCNK1 or K2P1), which was originally cloned from the human kidney^2^. However, the electrophysiological properties and functional roles of TWIK-1 are poorly understood because the TWIK-1 current cannot be measured in heterologous expression systems^3–7^.

The TWIK-1 channel is widely expressed in various tissues, including those of the heart, kidney, and brain^8,9^. In the brain, TWIK-1 mRNA is highly expressed in various types of neurons^10,11^; however, studies describing neuronal function of TWIK-1 are scarce. Deng et al.^12^ reported that serotonin inhibits the excitability of stellate and pyramidal neurons in the entorhinal cortex by activating TWIK-1. In addition, Plant et al.^5^ reported that TWIK-1 can form heterodimeric channels with TASK-1 (TWIK-related acid-sensitive K^+^ channel 1 or KCNK3) or TASK-3 (KCNK9), and these heterodimeric channels display acid-sensitive and halothane-sensitive outwardly rectifying K^+^ currents in cerebellar granule neurons. In the previous study, we showed that TWIK-1 proteins were expressed and localized mainly in the soma and proximal dendrites of dentate gyrus granule cells (DGGCs) and that TWIK-1-mediated outwardly rectifying K^+^ currents, contributing to the intrinsic excitability of DGGCs ^13^.

Interestingly, recent studies have shown that TWIK-1 can form heterodimeric channels in astrocytes and neurons with other isoforms of K2P family members, including TREK-1 (TWIK-related K^+^ channel 1 or KCNK2), TASK-1, and TASK-3^5,14,15^. Whereas TWIK-1/TREK-1 heterodimeric channels display a linear current–voltage (I–V) relationship in astrocytes^14,15^, TWIK-1/TASK-1 and TWIK-1/TASK-3 heterodimeric channels mediate outwardly rectifying K^+^ currents in cerebellar granule neurons^5^. TASK-3 is also highly expressed in the hippocampal region^10^ and is inhibited by the activation of neurotensin receptor 1 (NTSR1)^16^. Because TWIK-1-mediated currents exhibit outwardly rectifying K^+^ currents in the DGGCs of the hippocampus^13^, we hypothesized that TWIK-1 can also act as a functional K^+^ channel by forming a heterodimeric channel with TASK-3 (TWIK-1/TASK-3). We further hypothesized that because the resulting heterodimeric channel contains TASK-3, it would be regulated by NTSR1 signaling in DGGCs of the mouse hippocampus.

The aim of this study was to investigate the expression of functional TWIK-1/TASK-3 heterodimeric channels in DGGCs. We found that the TWIK-1/TASK-3 heterodimer contributed to the intrinsic excitability of DGGCs and that the firing frequency of DGGCs was increased via inhibiting TWIK-1/TASK-3 heterodimeric channels when applied with the neuromodulator neurotensin (NT).

## Materials and methods

### Chemicals

Bicuculline methobromide, CGP55845 hydrochloride, D-AP5, CNQX, QX314, and tetraethylammonium chloride (TEA) were purchased from Tocris Bioscience (Bristol, UK). 4-Aminopyridine (4-AP) was purchased from Sigma-Aldrich (St. Louis, MO, USA).

### Animals

Male C57BL/6 mice aged 7–8 weeks were used for the experiments. Animal care and handling were performed in accordance with the instructional guidelines of the Korea Institute of Science and Technology (Seoul, Korea) and Korea University (Seoul, Korea).

### Plasmids and small hairpin-forming interference RNA (shRNA)

cDNAs encoding full-length mouse TWIK-1 (NM_008430) and mouse TASK-3 (NM_001033876) were obtained by using the Gateway cloning method (Invitrogen, Carlsbad, CA, USA). cDNAs encoding full-length human NTSR-1 (NM_002531) and human NTSR-2 (NM_012344) were synthesized by designing gene blocks (gBlocks^®^ Gene Fragments; Integrated DNA Technologies, Coralville, IA, USA) and constructing entry clones using the Gateway BP cloning method (Invitrogen). The constructs were cloned into several vectors, including pDEST-HA-N, pDEST-FLAG-N, pDEST-IRES2-GFP, and pDEST-IRES2-mCherry by using Gateway LR cloning (Invitrogen). To construct the concatenated TWIK-1 and TASK-3, TASK-3 and TWIK-1 were recloned into the pDONR207 P1P5R and pDONR207 P5P2 vectors, respectively, via two independent BP reactions (Invitrogen), followed by a MultiSite Gateway LR recombination reaction (Invitrogen) according to the manufacturer’s guidelines to produce pDEST-IRES2-GFP. The target regions of the shRNA (mouse TWIK-1: 5′-GCATCATCTACTCTGTCATCG-3′; mouse TASK-3: 5′-GCTGGTGTCCAGTGGAAATTC-3′) were obtained by oligonucleotide-directed mutagenesis using an EZchange site-directed mutagenesis kit (Enzynomics, Daejeon, Korea).

### Construction of the recombinant adenovirus vector

To produce the recombinant adenovirus vector, U6-loxP-CMV-mCherry-shRNA sequences from pSicoR-Scrambled (Sc) shRNA, pSicoR-TWIK-1 shRNA^13,14^ and pSicoR-TASK-3 shRNA were cloned into pDONR^TM^ 207 vectors (Invitrogen) and confirmed by DNA sequencing. An LR recombination reaction was performed between the cloned plasmid and the pAD/CMV/V5-DEST^TM^ destination vector using LR Clonase^TM^ enzyme mix. Adenoviral vectors carrying each shRNA were linearized by PacI digestion and then transfected into HEK293A cells using Lipofectamine® 3000 reagent. Purification of adenovirus was performed according to the manufacturer’s protocol (ViraBind^TM^ Adenovirus purification kit, Cell Biolabs, Inc., San Diego, CA, USA).

### Bimolecular fluorescence complementation assay

For the bimolecular fluorescence complementation (BiFC) assay, TWIK-1, TASK-3, and GIRK-1 were cloned into the pBiFC-VN173 and pBiFC-VC155 vectors. COS-7 cells were co-transfected with the cloned BiFC vectors in all possible pairwise combinations and then transfected with DsRed-Mem for the detection of plasma membranes. The following day, these cells were fixed with 4% paraformaldehyde for 20 min at room temperature (RT) and then mounted with Dako Fluorescence Mounting Medium. Venus fluorescence signals were observed by confocal microscopy.

### Duolink proximity ligation assay

Protein–protein interactions between endogenous proteins were detected by using a Duolink proximity ligation assay (PLA) kit (Sigma-Aldrich). The PLA probe anti-Rabbit MINUS binds to the TASK-3 antibody; whereas, the PLA probe anti-Goat PLUS binds to the TWIK-1 antibody. After pre-incubation with a blocking agent for 1 h, the frozen sectioned hippocampal tissues were incubated overnight with primary antibodies to TASK-3 (1:100, Abcam, Cambridge, UK) and TWIK-1 (1:100, Santa Cruz Biotechnology, Dallas, TX, USA). Duolink PLA probes detecting rabbit or goat antibodies were diluted in the antibody diluent to a concentration of 1:5 and applied to the slides, followed by incubation for 2 h in a preheated humidity chamber at 37 °C. Duolink hybridization stock (dilution 1:5) was used to hybridize the two Duolink PLA probes, which were incubated in a preheated humidity chamber for 30 min at 37 °C and then incubated in a ligation solution consisting of Duolink ligation stock (1:5) and Duolink Ligase (1:40) for 90 min at 37 °C. Detection of the amplified probe was performed with the Duolink detection kit with the Duolink detection stock diluted at 1:5 and applied for 1 h at 37 °C. The final washing steps were performed in saline sodium citrate buffer.

### Single-cell reverse transcription-polymerase chain reaction (RT-PCR)

The expression patterns of TWIK-1, TASK-1, and TASK-3 in DGGCs were examined using single-cell RT-PCR assays with SuperScript III CellsDirect cDNA Synthesis kits (Invitrogen) according to the manufacturer’s manual. The expression of Calbindin1 was used as a marker for mature granule cells. The patch pipettes were filled with RNase-free internal solution. The cytoplasm was collected into a patch pipette and expelled into a PCR tube. The cDNA was synthesized according to the kit description using gene-specific antisense primers instead of oligo dT (Supplementary Table 1). Two rounds of PCR reactions were then performed using 2× TOPsimple DyeMIX-Tenuto (Enzynomics) with a C1000 thermal cycler (Bio-Rad, CA, USA). For the initial RT-PCR, all target genes were amplified together in one tube, and the PCR product was used as the template for the second round of PCR. The second round of PCR was performed individually for each gene. The PCR products were separated by electrophoresis in a 2% agarose gel, and images were captured on a gel imaging system.

### Immunohistochemistry

Following intracardiac perfusion with phosphate-buffered saline (PBS), brain slices were fixed overnight with 4% paraformaldehyde (v/v) and then incubated for 24 h with 30% sucrose in PBS. The brain tissues were stored at −80 °C in frozen section compound (Leica Biosystems). Frozen tissue blocks were sectioned into 30-µm-thick frozen sections using a cryostat. The sections were incubated with 0.5% Triton X-100 in PBS for 20 min at RT followed by blocking with 10% donkey serum and 0.1% Triton X-100 in PBS for 1 h at RT. The sectioned samples were incubated at 4 °C overnight with the primary antibody, either anti-rabbit KCNK-9 (TASK-3) (1:300, ab85289; Abcam) or chicken polyclonal anti-MAP2 (1:500, Abcam). After washing, Alexa 488- or 549-conjugated secondary antibodies (1:400, Jackson ImmunoResearch, West Grove, PA, USA) were added and incubated for 1 h at RT. The tissues were counterstained with DAPI, and the fluorescence labeling was analyzed using an A1 Nikon confocal microscope.

### Western blotting

The mouse brain was isolated, and the dentate gyrus and the rest of the hippocampal regions were dissected. The dentate gyrus tissues obtained were lysed with RIPA buffer and processed for western blotting using anti-TASK-3 antibody (1:1000, LifeSpan BioSciences, Seattle, WA, USA) and mouse anti-actin (1:2000, Sigma-Aldrich). Primary cultured astrocytes were used as a negative control. Signals were detected by enhanced chemiluminescence (GE Healthcare, Chicago, IL, USA) following probing with the appropriate horseradish peroxidase-conjugated secondary antibodies (1:3000, Jackson ImmunoResearch). Each experiment was performed with samples from three independent groups.

### Co-immunoprecipitation

HEK293T cells were transfected with HA-TWIK-1 and Flag-TASK-3 using polyethylenimine (PEI, Sigma-Aldrich). At 24 h after transfection, the cells were lysed with RIPA buffer composed of 50 mM Tris-Cl, 150 mM NaCl, 1% NP-40, 0.5% sodium deoxycholate, 0.1% sodium dodecyl sulfate (SDS), and protease inhibitor cocktail (Tech & Innovation, Kangwon, Korea) and incubated for 1 h at 4 °C. The cell lysates were centrifuged at 15,000×*g* for 30 min at 4 °C, and the supernatants were moved to new tubes. The lysates were incubated with anti-FLAG antibody (Sigma-Aldrich) on a rocking mixer overnight at 4 °C and then for an additional 1 h with Protein G Agarose (Santa Cruz Biotechnology). The immunocomplexes were rinsed three times with RIPA buffer and separated by SDS-polyacrylamide gel electrophoresis. The target proteins were transferred onto PVDF membranes and detected by using anti-HA (Roche, Basel, Switzerland) or anti-FLAG antibodies.

### Stereotaxic viral injection

Virus injections were performed on mice deeply anesthetized with Avertin (2,2,2-tribromethanol in 2-methyl butanol) and secured in a stereotaxic apparatus (Kopf Instruments, Tujunga, CA, USA). Briefly, the scalp was opened, and two holes were drilled in the skull. Ad-shTASK-3-mCherry, Ad-shTWIK-1-GFP, and Ad-Sc shRNA-mCherry were injected into the dentate gyrus through a glass micro dispenser (VWR, Radnor, PA, USA) using a syringe pump (KD Scientific, Holliston, MA, USA) at a speed of 0.2 µl/min. The coordinates were as follows: −2.0 mm anteroposterior, ±1.3 mm lateral, and 1.6 mm dorsoventral from the dura. The micro dispenser was put in place for 2 min before the injection and left for 2 min after the injection.

### Slice preparation and electrophysiology

Brain slice electrophysiology was performed at 2 weeks after virus injections. Mice were decapitated after induction of anesthesia by Halothane (2-bromo-2-chloro-1,1,1-trifluoroethane). Horizontal 400-µm-thick hippocampal brain slices were prepared using a vibratome (Linear Slicer Pro7, D.S.K, Japan) in ice-cold oxygenated artificial cerebrospinal fluid containing (in mM) 130 NaCl, 2.5 KCl, 1.25 KH_2_PO_4_, 3.0 MgCl_2_, 1.0 CaCl_2_, 26 NaHCO_3_, and 10.0 d-glucose. The slices were stored at RT until the experiments were performed. The virus-injected hippocampal slices were visualized using an Olympus BX51WI microscope equipped with epifluorescence. The mCherry-positive cells were selected for whole-cell patch clamp recording. Patch pipettes were fabricated from borosilicate glass and had a resistance of 7–10 MΩ. The pipette solution contained (in mM) 120 potassium gluconate, 10 KCl, 1 MgCl_2_, 0.5 EGTA, and 40 HEPES (with the pH adjusted to 7.2 with KOH).

In the voltage clamp experiments, the holding membrane potential was set at −70 mV, and series and input resistances were monitored throughout the experiment using a −5-mV pulse. Recordings were considered stable when the series and input resistance and the RMP did not vary by >20%. Recordings were filtered at 2 kHz and digitized at 10 kHz. In the current clamp experiments, a current was applied at increasing stepwise increments of 5 pA, each with a duration of 1.2 s. These experiments were performed in the presence of the GABA_A_ and GABA_B_ antagonists bicuculline (10 µM) and CGP 55845 (10 µM). The data were collected with a Multi-Clamp 700B amplifier (Molecular Devices, Sunnyvale, CA, USA) using Clampex10 acquisition software (Molecular Devices) and digitized with a Digidata 1322 A (Molecular Devices). The experiments were performed at RT.

### Electrophysiological recording in COS-7 cells

COS-7 cells maintained in RPMI medium supplemented with 10% fetal bovine serum were plated onto coverslips for electrophysiology experiments. The pipette solution contained (in mM) 150 KCl, 1 CaCl_2_, 1 MgCl_2_, 5 EGTA, and 10 HEPES (with the pH adjusted to 7.2 with KOH). The bath contained, in mM, 150 NaCl, 3 KCl, 2 CaCl_2_, 1 MgCl_2_, 10 HEPES, 5.5 D-glucose, and 20 sucrose (with the pH adjusted to 7.4 with NaOH). The patch pipettes were fabricated from borosilicate glass capillaries (Warner Instruments, Inc., Hamden, CT, USA) and had a resistance of 5–6 MΩ. Whole-cell currents were recorded using a patch clamp amplifier (Axopatch 700B, Axon Instrument, Inc., Norwood, MA, USA). The *I–V* relationship was measured by applying ramp pulses (from −120 mV to +40 mV over a period of 1000 ms) from a holding potential of −60 mV. A Digidata 1550 A interface (Molecular Devices) was used for digital–analog signal conversion between the amplifier and computer. The data were sampled at 5 kHz and filtered at 1 kHz. The currents were analyzed with Clampfit software (Axon Instruments, Inc.). The experiments were conducted at RT.

### Statistical analysis

All statistical analyses were performed using GraphPad Prism version 5.00 for Windows. Numerical data are presented as the means ± standard error of the mean (SEM). The error bars in the graphs denote the SEM. The statistical significance of the data was assessed by unpaired or paired Student’s *t*-tests, with the significance level denoted by asterisks (*P < 0.05, **P < 0.01, or ***P < 0.001).

## Results

### TWIK-1 and TASK-3 are coexpressed in mouse hippocampal DGGCs

**Fig. 1:**
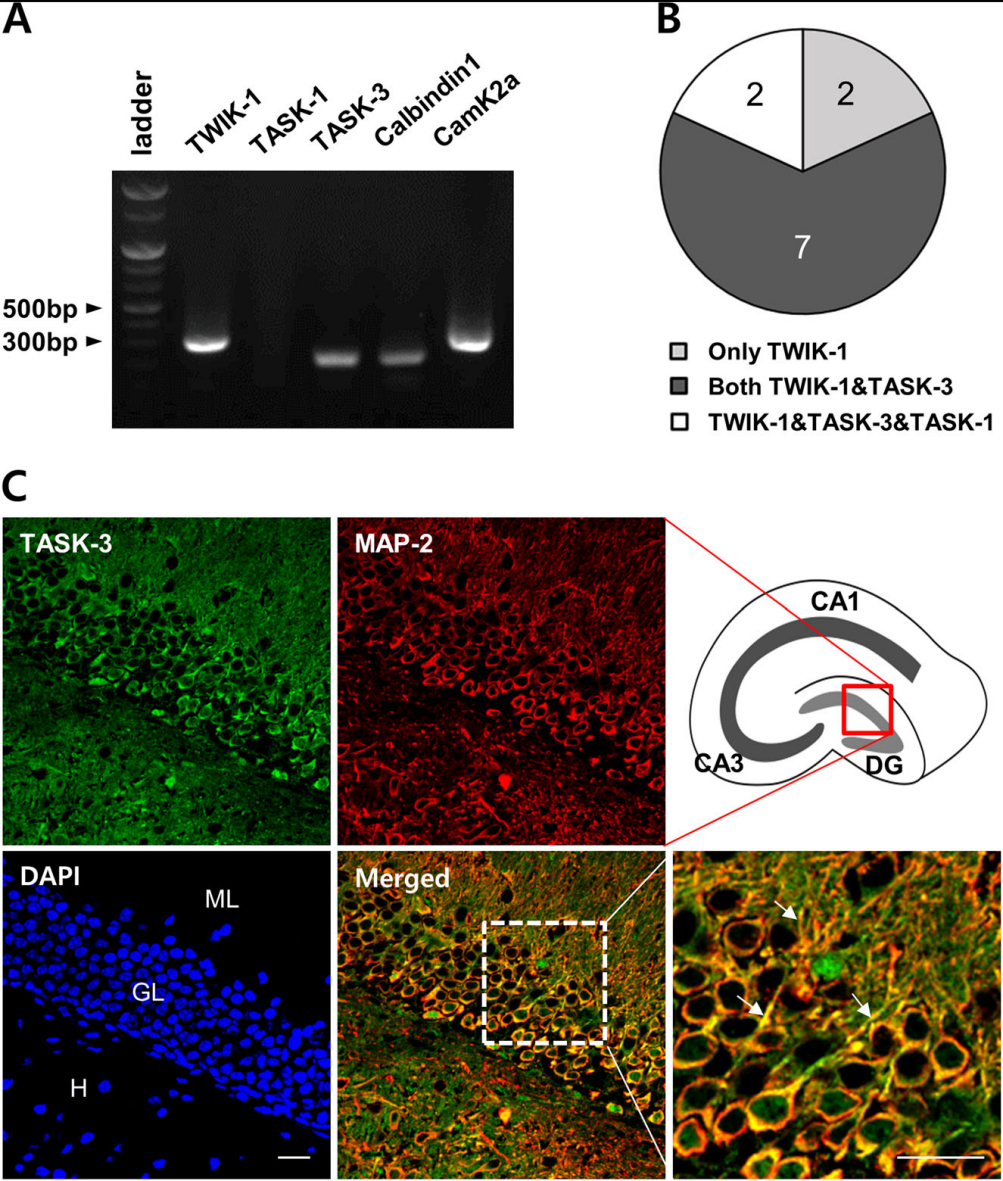
TWIK-1 and TASK-3 are coexpressed in mouse hippocampal DGGCs. **a** Representative electrophoresis image of single-cell RT-PCR showing the expression of TWIK-1, TASK-1, TASK-3, Camk2α, and Calbindin1 in single DGGC. **b** A pie chart showing proportion of TWIK-1, TASK-1, and TASK-3 expression in the DGGCs. **c** Representative fluorescence images of the dual immunostaining of TASK-3 (green) and MAP-2 (red), with DAPI staining of the nuclei (blue) of dentate gyrus granule cells in the mouse hippocampus. The images show that TASK-3 was highly expressed in the dentate granular cell layer. The merged image demonstrates that TASK-3 was co-localized with MAP-2-positive proximal dendrites of granular cells (Magnified image, arrows). Scale bar, 20 μm. ML molecular layer, GL granule layer, H hilus, CA1 cornu ammonis 1, CA3 cornu ammonis 3, DG dentate gyrus

Previous studies have demonstrated outwardly rectifying K^+^ currents of TWIK-1/TASK-1 and TWIK-1/TASK-3 heterodimeric channels in cerebellar granule cells^5^, as well as TWIK-1-mediated outwardly rectifying K^+^ currents in mouse hippocampal DGGCs^13^. Based on these findings, we first investigated whether TASK-1 or TASK-3 was coexpressed with TWIK-1 in the same mouse hippocampal DGGCs using single-cell RT-PCR. The cytoplasm of DGGCs was collected with patch pipettes, and the TWIK-1, TASK-1, and TASK-3 genes were amplified. We found that all examined neurons expressed the neuronal maker CamK2α and the mature DGGC marker Calbindin1 (Fig. 1a)^17,18^. The single-cell RT-PCR results showed that among DGGC neurons tested, 100% (11 of 11) expressed TWIK-1, 82% (9 of 11) expressed TASK-3, and 18% (2 of 11) expressed TASK-1 (Fig. 1b). These data suggested that TASK-3 might be a strong candidate for a heterodimeric partner with TWIK-1 in mature hippocampal DGGCs.

To further confirm the expression and localization of TASK-3 proteins in DGGCs, we performed an immunohistochemistry analysis using antibodies against mouse TASK-3 and MAP-2, a dendritic marker. The results demonstrated that TASK-3 proteins were highly localized in the granular and molecular layers of DGGCs and that TASK-3 and MAP-2 were co-localized in the somata and proximal dendrites of DGGCs (Fig. 1c). Collectively, these results confirmed the coexpression of TWIK-1 mRNA and TASK-3 mRNA in DGGCs and demonstrated the expression pattern of TASK-3 proteins in the somata and proximal dendrites of DGGCs. Because, TWIK-1 proteins are also localized mainly in the somata and proximal dendrites of DGGCs^13^, it is plausible that TASK-3 can form heterodimeric channels with TWIK-1 in DGGCs.

### TWIK-1 is associated with TASK-3 in COS-7 cells and DGGCs

**Fig. 2:**
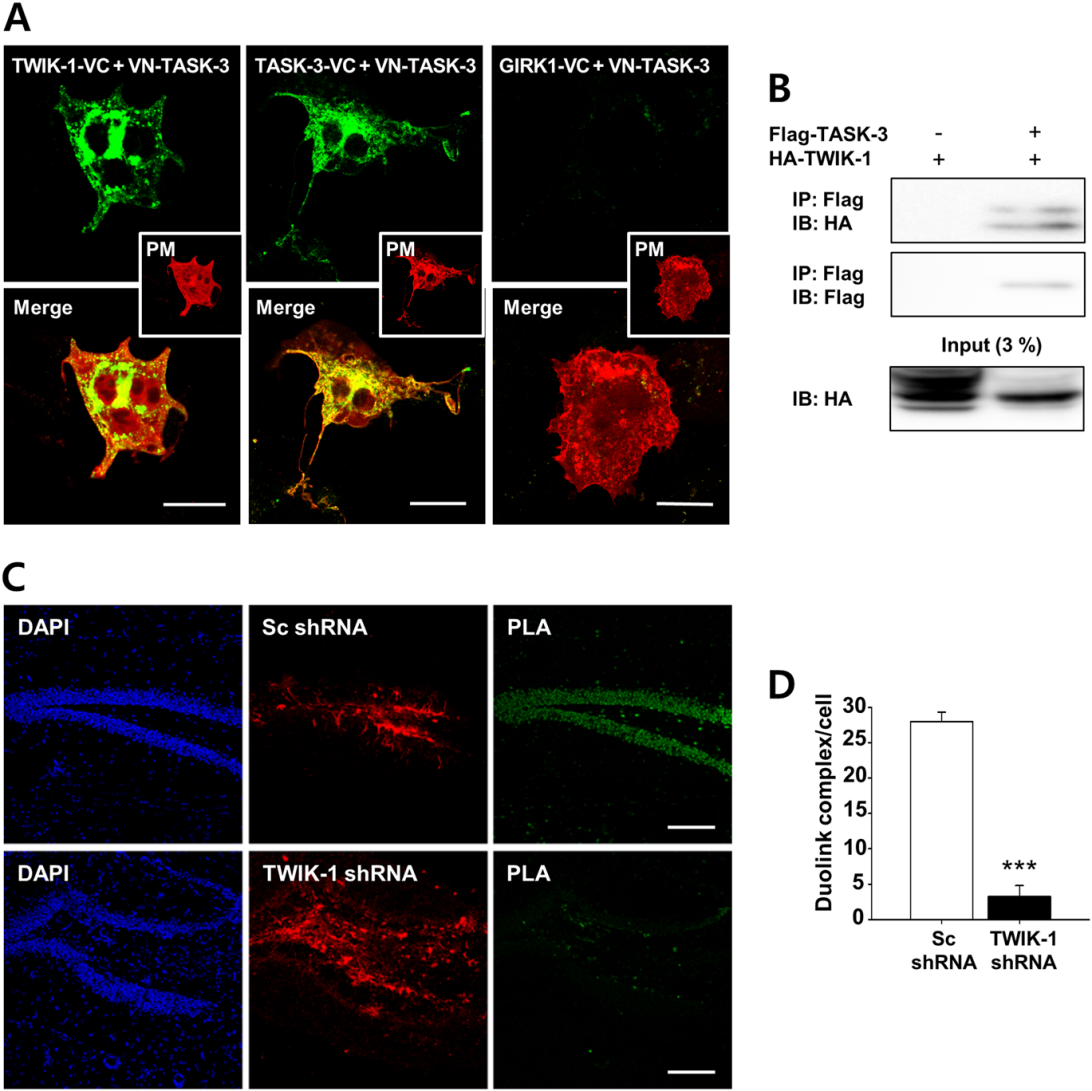
TWIK-1 forms a heterodimeric channel with TASK-3 in the heterologous system and DGGCs. **a** Representative fluorescence images of the bimolecular fluorescence complementation assay. VN and VC are the N-terminal and C-terminal fragments of the Venus, respectively. Intense Venus signals (green) were examined for dimerization. TWIK-1-VC strongly interacted with VN-TASK-3. The TASK-3 homodimer was used as a positive control, and the GIRK1-VC and VN-TASK-3 heterodimer was used as a negative control (upper). The red images represent the plasma membrane by DsRed-Mem (middle). The merged images showed that TWIK-1 and TASK-3 heterodimers were located in the plasma membrane (yellow color, lower). Scale bar, 10 µm. **b** The lysates from HEK293T cells transfected as described were subjected to immunoprecipitation with an anti-Flag antibody, and the immunocomplexes were analyzed by immunoblotting with an anti-HA antibody. TWIK-1 coprecipitated only in the presence of TASK-3 (top panel). The expression of these two channels is shown in the lower panels. **c** Representative images of the Duolink proximity ligation assay in DGGCs infected with Ad-Sc or Ad-TASK-3 shRNAs. Scale bar, 50 μm. **d** Quantification of the Duolink spots in DGGCs expressing Ad-Sc or Ad-TASK-3 shRNAs. ***P *<* 0.001

Next, we performed BiFC assays to examine the interaction between TWIK-1 and TASK-3 at the single-cell level. This allowed visualization of the two independent proteins in close spatial proximity^16,19,20^. We constructed variants of TWIK-1 and TASK-3 in which the N- or C-terminus was fused to the complementary half of a split Venus fluorescent protein [i.e., the C-terminal half (VC) or the N-terminal half (VN), respectively]; both were then transfected into COS-7 cells (Fig. 2a). When the split Venus halves were in complementary positions on each subunit (TWIK-1-VC + VN-TASK-3), strong fluorescence was detected (Fig. 2a, left panel). TASK-3 homodimers (TASK-3-VC + VN-TASK-3) were used as a positive control and showed strong BiFC signals as expected (Fig. 2a, middle panel). By contrast, when the BiFC assay was performed with the TASK-3 and GIRK1 K^+^ channels as a negative control, no complementation (Fig. 2a, right panel). We then examined the physical interaction between TWIK-1 and TASK-3 in a mammalian system. We constructed expression vectors for Flag-tagged TASK-3 (Flag-TASK-3) and hemagglutinin (HA)-tagged TWIK-1 (HA-TWIK-1) and coexpressed them in HEK293T cells. Cell lysates were co-immunoprecipitated with an anti-Flag antibody, and blotting was performed with an anti-HA antibody. The results showed a clear association between HA-TWIK-1 and Flag-TASK-3 (Fig. 2b).

Next, we investigated whether endogenous TWIK-1 was associated with endogenous TASK-3 in DGGCs by performing a Duolink PLA in DGGCs infected with an adenovirus (Ad) carrying a shRNA targeted against TWIK-1 (Ad-TWIK-1 shRNA) or Ad-Sc shRNA. The specificity of the Ad-TWIK-1 shRNA we used has previously been documented^13,14^. We found that DGGCs expressing the Sc shRNA showed a strong PLA signal from TWIK-1 and TASK-3, but no such signal was detected in DGGCs expressing TWIK-1 shRNA (Fig. 2c). PLA signals were quantified as shown in Fig. 2d. As a negative control, a Duolink PLA was performed for a mouse fibroblast cell line, NIH3T3, in which K2P is poorly expressed. There was no positive PLA signal in these cells compared with native DGGCs (data not shown). These results strongly suggested a physical association between TWIK-1 and TASK-3 in DGGCs, providing evidence for the formation of TWIK-1/TASK-3 heterodimeric channels in these cells.

### TWIK-1/TASK-3 heterodimeric channels contribute to K^+^ conductance in DGGCs

The strong PLA signal from TWIK-1 and TASK-3 in DGGCs indicated that TWIK-1/TASK-3 heterodimeric channels contributed to the electrical properties of DGGCs. To assess the function of the TWIK-1/TASK-3 channel in DGGCs, we first examined the effect of knocking down TWIK-1 and TASK-3 on the whole-cell currents in these cells. We constructed a new shRNA targeted against mouse TASK-3 (TASK-3 shRNA) and validated its knockdown efficiency by western blot analysis (Supplementary Figure 1A). We also developed an Ad carrying TASK-3 shRNA (Ad-TASK-3 shRNA). This virus also contained DNA encoding the mCherry fluorescence marker to allow visualization of the location and amount of the viral injection. Immunohistochemical staining performed 1 week after the injection of Ad-TASK-3 shRNA or Ad-Sc shRNA into the hippocampal dentate gyrus of mouse brain showed that the expression of TASK-3 in DGGCs was remarkably reduced by Ad-TASK-3 shRNA infection (Supplementary Figure 1B).

The contribution of TASK-3 to the electrophysiological properties of mature DGGCs was assessed by a comparative analysis of the whole-cell I–V relationship in cells expressing Sc shRNA or TASK-3 shRNA. Electrophysiological experiments were performed in the outer granule cell layer, in which mature DGGCs are mostly present. Several electrophysiological parameters of the examined DGGCs were similar to previously reported values of fully mature DGGCs (Table 1)^21^. To isolate the K2P component of the whole-cell currents, we pharmacologically suppressed inwardly rectifying and voltage-gated K^+^ currents using a combination of 1 mM Cs^+^ (a standard blocker of inwardly rectifying K^+^ channels), 5 mM TEA and 5 mM 4-AP (blockers of voltage-gated K^+^ channels). The administration of Cs^+^/TEA/4-AP (1 mM Cs^+^, 5 mM TEA, and 5 mM 4-AP) caused a marked reduction in both outward and inward currents (by 50.7 ± 1.1% and 63.7 ± 3.3% at holding potentials of +50 mV and −150 mV, respectively; Supplementary Figure 2A and 2B). The Sc shRNA did not significantly affect the total K^+^ currents and Cs^+^/TEA/4-AP-insensitive currents (80.67 ± 3.8 pA/pF and 40.39 ± 3.4 pA/pF at +50 mV, respectively; Supplementary Figure 2C and 2D).

**Table 1:** Electrical properties of DGGCs

	Naive (*n* = 35)	Sc shRNA (*n* = 27)	TASK-3 shRNA (*n* = 29)	TASK-3 shRNA + TWIK-1 shRNA (*n* = 38)
Input resistance (MΩ)	210.24 ± 11.22	212.33 ± 13.08	232.71 ± 9.05	234.24 ± 10.61
Resting membrane potential (mV)	−75.15 ± 0.31	−75.01 ± 0.97	−73.82 ± 0.64*	−73.97 ± 0.29*
Membrane capacitance (pF)	53.27 ± 1.64	54.67 ± 1.89	56.06 ± 1.55	52.39 ± 1.96
Number of spikes (at 105 pA)	3.10 ± 0.34	2.75 ± 0.31	19.10 ± 0.66***	20.00 ± 0.56***
AP threshold (mV)	−35.02 ± 0.91	−35.39 ± 0.81	−38.27 ± 0.72**	−38.75 ± 0.74**

The data are presented as mean ± SEM. Statistical analyses were performed by two-tailed *t*-tests

*p < 0.05, **p < 0.01, ***p < 0.001

**Fig. 3:**
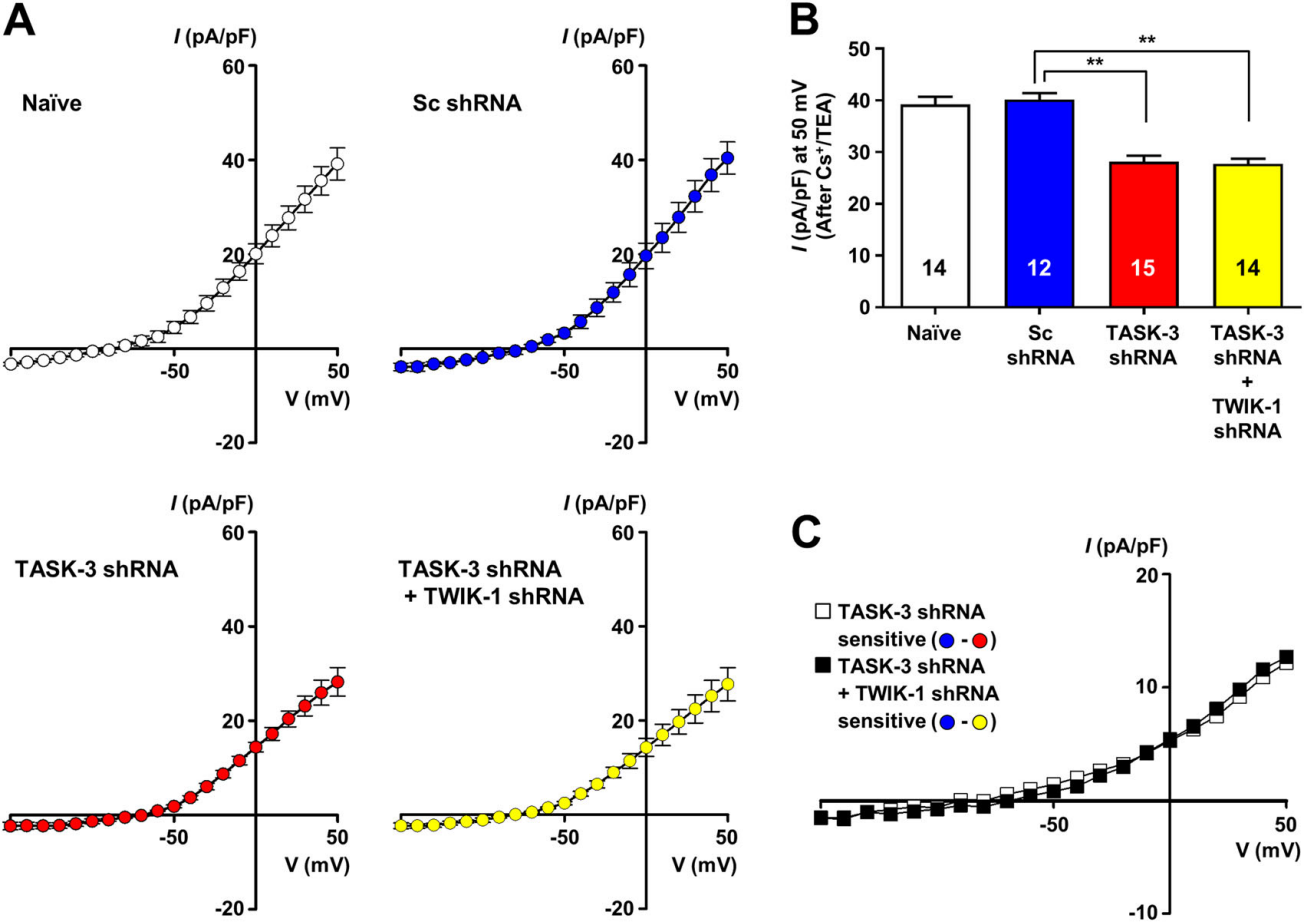
TWIK-1/TASK-3 heterodimeric channels contribute to K^+^ conductance in DGGCs. **a** The averaged current*–*voltage (I–V) relationship of the whole-cell currents from DGGCs infected with Ad-Sc or Ad-TASK-3 shRNAs or both Ad-TASK-3 shRNA and TWIK-1 shRNA as well as from naive DGGCs was measured in standard artificial cerebrospinal fluid in the presence of Cs^+^/TEA/4-AP (1 mM/5 mM/5 mM). Whole-cell currents were elicited by 1-s-duration ramp pulses descending from 50 mV to −150 mV from a holding potential of −70 mV. **b** A summary bar graph for **a**. The mean values of the current density in naive DGGCs (*n* = 14 cells, *N* = 4 mice) or DGGCs expressing Sc shRNA (*n* = 12 cells, *N* = 3 mice), TASK-3 shRNA (*n* = 15 cells, *N* = 3 mice), or both TASK-3 and TWIK-1 shRNAs (*n* = 15 cells, *N* = 3 mice) measured in the presence of Cs^+^/TEA/4-AP (1 mM/5 mM/5 mM) are shown. The current density values are depicted at +50 mV. **c** The TASK-3 shRNA- or both TASK-3 shRNA- and TWIK-1 shRNA-sensitive currents were determined by subtracting each of the shRNA averaged currents from the Sc shRNA averaged currents **a**. **P < 0.01

Cs^+^/TEA/4-AP-insensitive currents in naive and Sc shRNA-infected DGGCs exhibited a prominent outwardly rectifying I–V relationship with a current density of 39.15 ± 3.38 pA/pF and 40.39 ± 3.4 pA/pF at +50 mV, respectively (Figs. 3a, b). However, the expression of TASK-3 shRNA in DGGCs significantly reduced the Cs^+^/TEA/4-AP-insensitive outward current in DGGCs (28.22 ± 3.04 pA/pF at +50 mV; Fig. 3a, b). Interestingly, there was no additional decrease in Cs^+^/TEA/4-AP-insensitive currents in TWIK-1 and TASK-3 double-knockdown cells (27.71 ± 3.5 pA/pF at 50 mV) compared to the effect of TASK-3 shRNA alone (Fig. 3a–c). To exclude the possible blockage of TASK-3 or TWIK-1/TASK-3 heterodimer channels by high concentrations of voltage-gated K^+^ channel blockers (Cs^+^/TEA/4-AP), we measured the effects of Cs^+^/TEA/4-AP on the overexpressed TASK-3 or TWIK-1/TASK-3 concaternated channels in COS-7 cells. Supplementary Figures 2C and 2D showed that the outward currents of the TASK-3 or TWIK-1/TASK-3 channels were not changed by treatment with K^+^ channel blockers, implying that most of the reduction of the Cs^+^/TEA/4-AP-insensitive currents shown in Fig. 3 was mediated by a deficiency of TWIK-1/TASK-3 heterodimeric channels. Taken together, these results suggested that TWIK-1 and TASK-3 can act together as a TWIK-1/TASK-3 heterodimeric channel and contribute to K^+^ conductance in DGGCs.

### TWIK-1/TASK-3 heterodimeric channels contribute to the intrinsic excitability of DGGCs

**Fig. 4:**
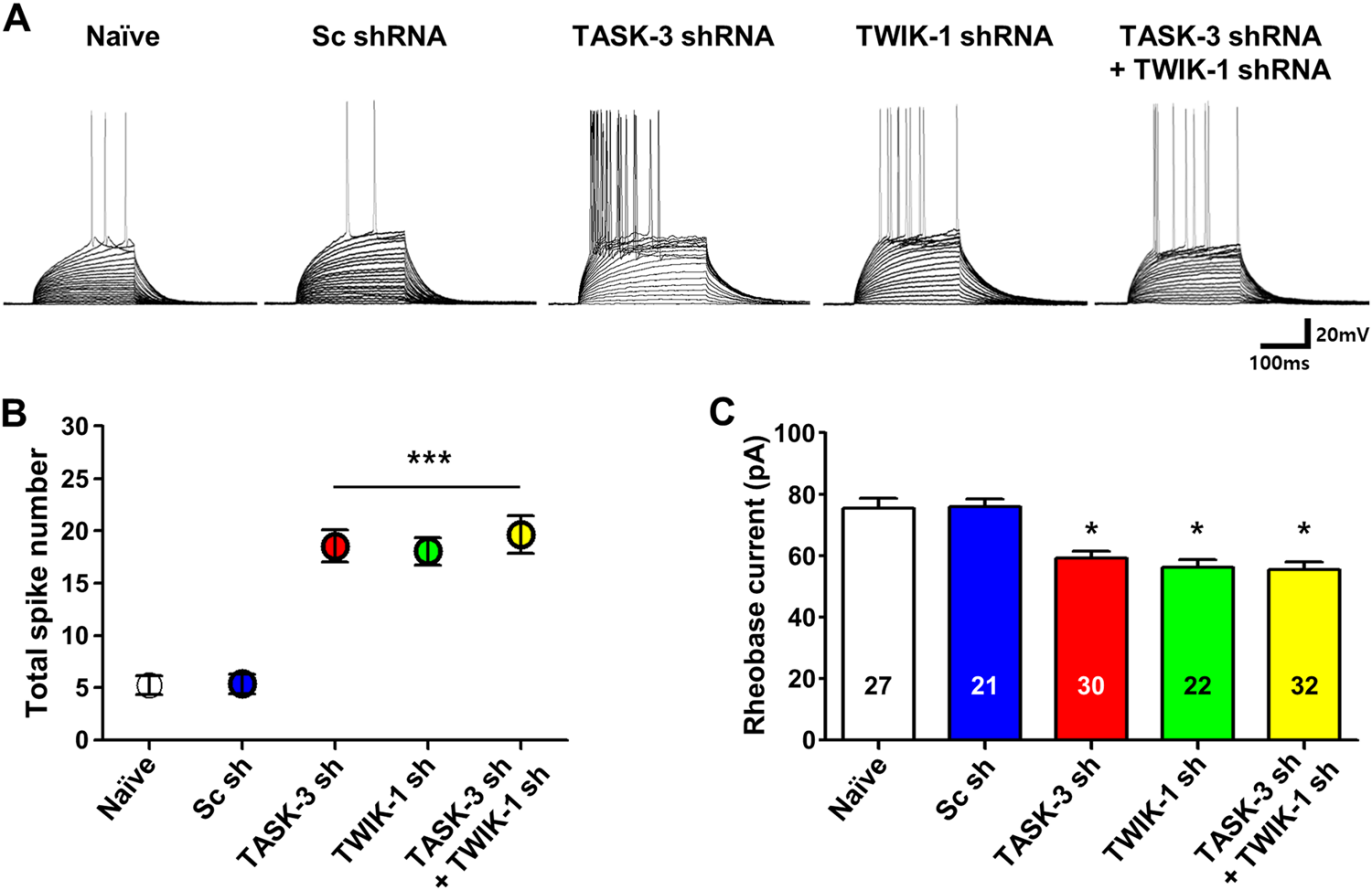
TWIK-1/TASK-3 heterodimeric channels contribute to the intrinsic excitability of DGGCs. **a** Representative traces of the membrane potential to stepwise current injections recorded from naive DGGCs (*n* = 27, *N* = 3) or DGGCs infected with Ad-Sc shRNA (*n* = 21, *N* = 3), Ad-TWIK-1 shRNA (*n* = 30, *N* = 3), Ad-TASK-3 shRNA (*n* = 22, *N* = 3), or both Ad-TWIK-1 shRNA and Ad-TASK-3 shRNA (*n* = 32, *N* = 3). The resting membrane potentials of the cells was maintained at −70 mV by constant current injections, and the depolarizing current was then injected stepwise in 5-pA increments. **b** The number of spikes indicated that the neuron infected with Ad-TWIK-1 shRNA and Ad-TASK-3 shRNA were more excitable compared to control mice. The recordings were performed in artificial cerebrospinal fluid containing 50 µM D-AP5, 10 µM CNQX, 10 µM bicuculline, 10 µM CGP 55845, 2 mM TEA, and 0.5 mM NiCl_2_, with a pipette solution containing 5 mM QX314. **c** Averaged values of rheobase currents in naive cells (*n* = 27 cells, *N* = 3 mice) and cells expressing Ad-Sc shRNA (*n* = 21 cells, *N* = 3 mice), Ad-TWIK-1 shRNA (*n* = 30 cells, *N* = 3 mice), Ad-TASK-3 shRNA (*n* = 12), or both Ad-TWIK-1 shRNA and Ad-TASK-3 shRNA (*n* = 32 cells, *N* = 3 mice). All values are means ± SEM. *P < 0.05, ***P < 0.001

A lack of TWIK-1-mediated K^+^ conductance causes augmented excitability of DGGCs resulting from a depolarized RMP or the lack of shunting neuronal excitation due to the absence of repolarizing outwardly rectifying K^+^ current^13^. To evaluate the role of TWIK-1/TASK-3 heterodimeric channels in the intrinsic excitability of DGGCs, we analyzed the firing rate of DGGCs by counting the numbers of action potentials evoked by depolarizing current injections. The firing rate of cells with downregulated TWIK-1 or TASK-3 was significantly higher than that of naive cells or control cells expressing Sc shRNA (Table 1, Fig 4a, b), providing evidence that TASK-3 and TWIK-1 contribute to the intrinsic excitability of DGGCs. Notably, double knockdown of TWIK-1 and TASK-3 did not have any significant additive effect on the firing rate of DGGCs.

We measured rheobase currents to further assess the excitability of DGGCs deficient in TWIK-1, TASK-3, or both channels (Fig. 4c, Table 1). The rheobase currents of TWIK-1 and TASK-3 double-knockdown DGGCs were similar to those of cells deficient in either TWIK-1 or TASK-3 alone. The RMP of the cells was maintained at −70 mV by a constant current injection. The rheobase currents were significantly smaller in the DGGCs deficient in TASK-3 or TWIK-1/TASK-3 than in the naive or Sc shRNA control cells (59.1 ± 2.4, 56.4 ± 2.5, 75.6 ± 3.2, and 76.1 ± 2.5 pA, respectively; Fig. 4c). Action potential (AP) thresholds were lower in DGGCs deficient in TASK-3 or TWIK-1/TASK-3 than in the naive DGGCs or DGGCs expressing Sc shRNA (−38.3 ± 0.7 mV, −38.8 ± 0.7 mV, −35.02 ± 0.9, and −35.4 ± 0.8, respectively; Table 1). Taken together, these results strongly suggested that TWIK-1 and TASK-3 function as subunits of TWIK-1/TASK-3 heterodimeric channels in DGGCs.

### TWIK-1/TASK-3 heterodimeric channels are inhibited by NT–NTSR1 signaling and contribute to the NT-induced increase in neuronal excitability in DGGCs

**Fig. 5:**
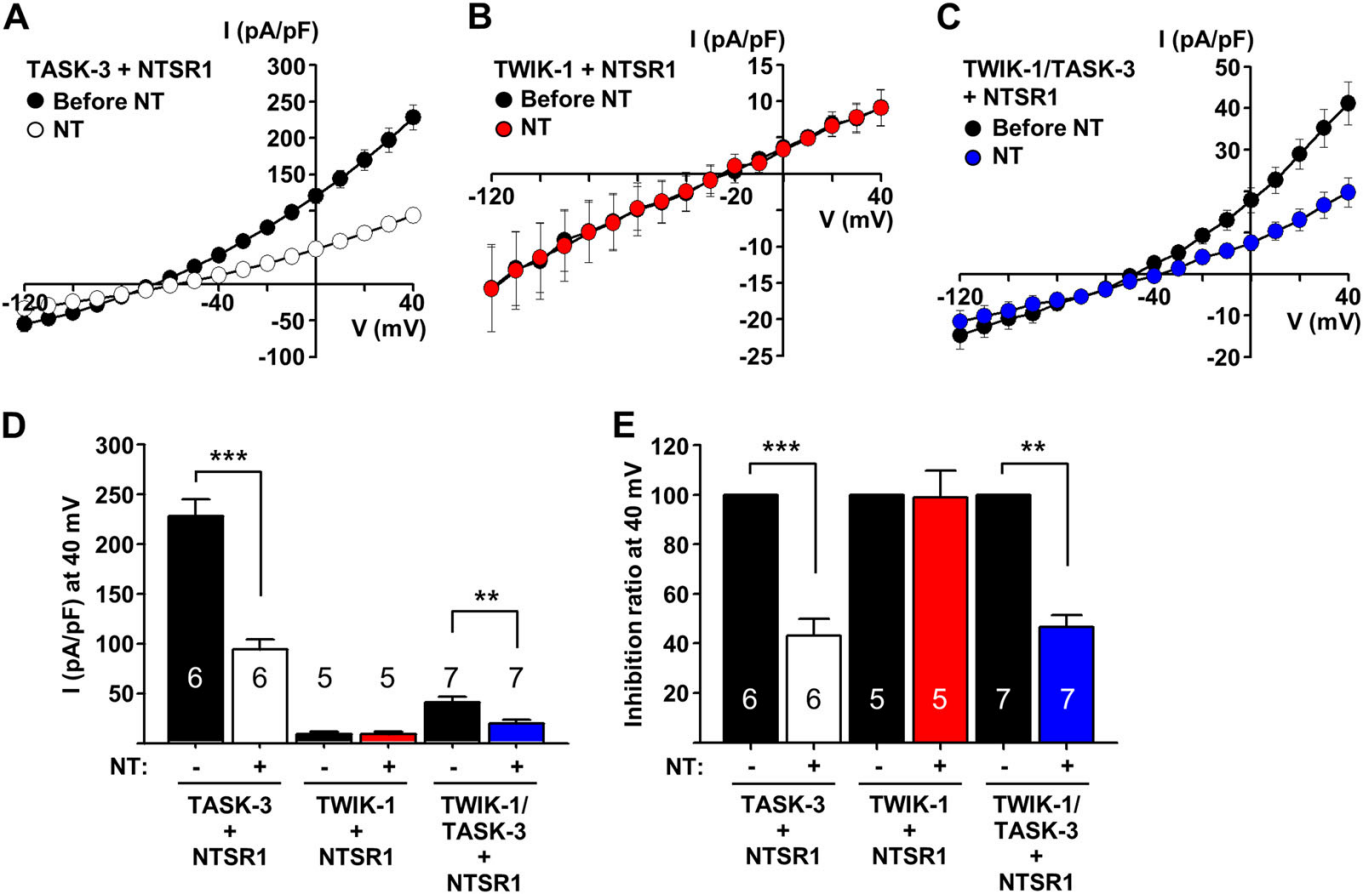
The activity of the TWIK-1/TASK-3 concatenated chimera is inhibited by NT–NTSR1 signaling. **a**–**c** Averaged I–V relationships in COS-7 cells transfected with TASK-3 and NTSR1 **a**, TWIK-1 and NTSR1 **b**, or the TASK-3/TWIK-1 concatenated channel and NTSR1 **c**, measured before and after the application of 10 μM neurotensin (NT) under a whole-cell configuration. **d** Summary bar graph showing normalized current density (as in **a**–**c**) at +40 mV. All values are means ± SEM (**P < 0.01 and ***P < 0.001). **e** Summary bar graph showing the inhibition ratio by NT application. All values are means ± SEM (**P < 0.01 and ***P < 0.001). The *P*-values were obtained with Student’s *t*-test

A previous study have shown the inhibition of TASK-3 channels by treatment with NT results in an increase in the neuronal excitability of DGGCs^18^. Because the activities of K2P heterodimeric channels, such as TREK-1/TREK-2, TASK-1/TASK-3 or TWIK-1 containing heterodimeric channels can be affected by regulatory processes known to affect each subunit^5,16,22,23^, it is possible that the TWIK-1/TASK-3 heterodimeric channels are regulated by NT–NTSR1 signaling. To determine whether NT can modulate the activity of TWIK-1/TASK-3 heterodimeric channels, we expressed several K2P channel constructs (TWIK-1, TASK-3, and a concatenated TWIK-1/TASK-3 heterodimeric channel) with NTSR1 in COS-7 cells. Consistent with previous results^16^, the activity of TASK-3 was inhibited by NT treatment (Fig. 5a, d and e). In addition, the cells expressing the concatenated TWIK-1/TASK-3 channel showed outwardly rectifying currents; these currents were significantly inhibited by NT treatment (Fig. 5c–e), whereas the activity of TWIK-1 was not affected (Fig. 5b, d and e). As a negative control experiment, we examined the effect of NT treatment on cells that expressed K2P channels (TWIK-1, TASK-3) alone or NTSR1 alone, which showed that NT did not affect the whole-cell currents of these transfected cells (Supplementary Figure 3). These results clearly indicated that the activity of TWIK-1/TASK-3 heterodimeric channels could be inhibited by the activation of NT–NTSR1 signaling.

**Fig. 6:**
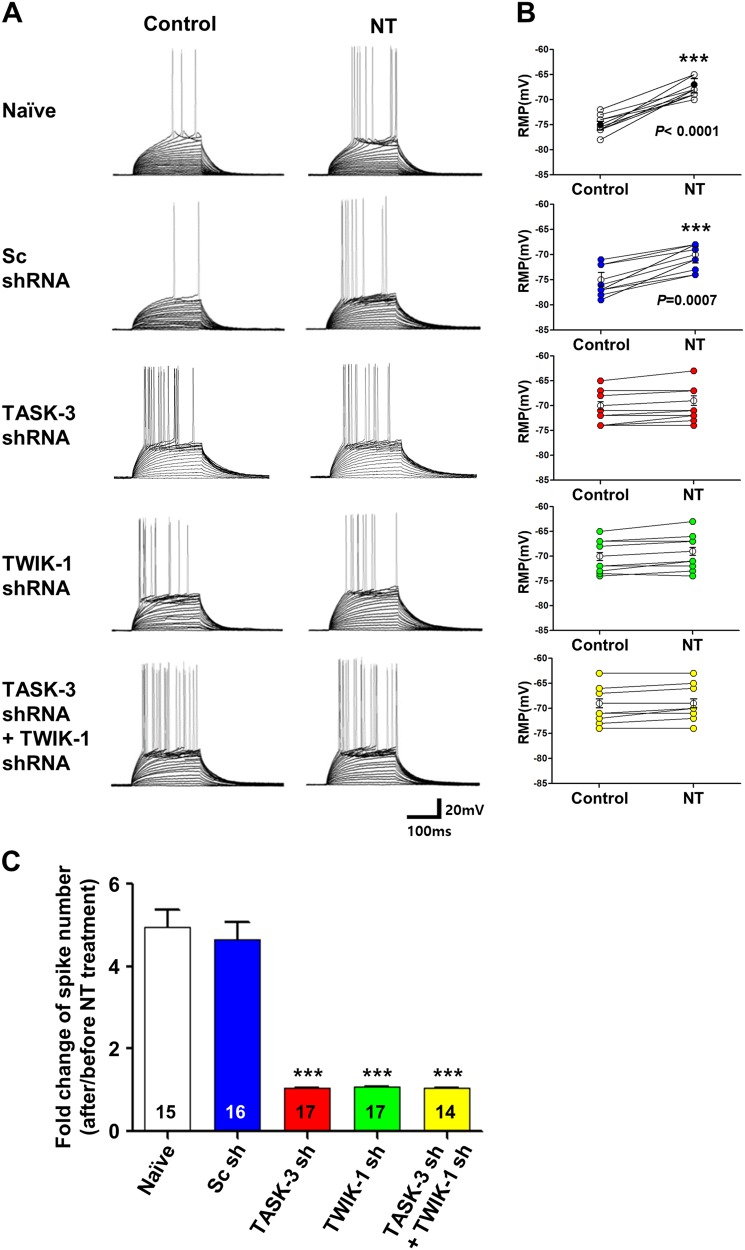
The NT-induced increase in neuronal excitability is mediated by inhibiting TWIK-1/TASK-3 heterodimeric channels. **a** Bath application of NT-mediated membrane depolarization of granule cells. A representative response of the membrane potential to stepwise current injections was recorded from naive DGGCs (*n* = 15, *N* = 3) or DGGCs infected with Ad-Sc shRNA (*n* = 16, *N* = 3), Ad-TWIK-1 shRNA (*n* = 17, *N* = 3), Ad-TASK-3 shRNA (*n* = 15, *N* = 3), or both Ad-TWIK-shRNA and Ad-TASK-3 shRNA (*n* = 14, *N* = 3). The resting membrane potential of these cells was maintained at −70 mV by constant current injections, and depolarizing currents were then injected stepwise at 5-pA increments until the membrane potential reached the firing threshold. **b**, **c** Analyzed bar charts of spike numbers at 105 pA for **a**. **b** Resting membrane potential values of the whole-cell currents in naive dentate gyrus granule cells (*n* = 15 cells, *N* = 3 mice) and cells expressing Sc shRNA (*n* = 16 cells, *N* = 3 mice), TWIK-1 shRNA (*n* = 17 cells, *N* = 3 mice), TASK-3 shRNA (*n* = 15 cells, *N* = 3 mice), or TWIK-1/TASK-3 shRNAs (*n* = 14 cells, *N* = 3 mice). **c** The extent of changes in the number of spikes following NT application in naive and Ad-Sc shRNA-infected DGGCs was larger than those in DGGCs transfected with Ad-TASK-1 shRNA alone, Ad-TWIK-1 shRNA alone, or both Ad-TWIK-1 shRNA and Ad-TASK-3 shRNAs. The data for Ad-TASK-3 shRNA alone, Ad-TWIK-1 shRNA alone, and both Ad-TWIK-1 shRNA and Ad-TASK-3 shRNAs showed no change in the numbers of spikes. The recordings were obtained in artificial cerebrospinal fluid containing 50 µM D-AP5, 10 µM CNQX, 10 µM bicuculline, 10 µM CGP55845, 2 mM TEA, and 0.5 mM NiCl_2_, with a pipette solution containing 5 mM QX314. All the data are presented as the means ± SEM. ***P < 0.001 was considered statistically significant

Next, to determine whether TWIK-1/TASK-3 heterodimeric channels were responsible for the NT-mediated firing enhancement of the DGGCs in vivo^16,24^, we directly measured the effects of knocking down TWIK-1 or TASK-3 in vivo by injecting Ad-TWIK-1 shRNA or Ad-TASK-3 shRNA in DGGCs. The firing rates of the action potential and RMP were then measured in DGGCs of hippocampal slices prepared from injected mice (Fig. 6a, b). Treatment with NT prominently increased the firing rates and depolarized the RMP of the naive or control Sc shRNA-infected DGGCs. Compared with its effects on naive or control Sc shRNA-infected DGGCs, the effect of NT treatment on the firing rates of the action potential and RMP clearly disappeared in DGGCs infected by Ad-TWIK-1 shRNA or Ad-TASK-3 shRNA. The double knockdown of TWIK-1 and TASK-3 in DGGCs did not show any additive effect. We also analyzed the fold change in the spike number after the final current injection. The extent of change in neuronal excitability induced by NT treatment was larger in naive and control Sc shRNA-infected DGGCs than in DGGCs infected with TWIK-1 shRNA alone, TASK-3 shRNA alone, or both shRNAs (Fig. 6c). These results indicated that TWIK-1/TASK-3 heterodimeric channels were responsible for the enhancement of firing rates and the depolarization of RMPs in hippocampal DGGCs induced by the application of NT.

## Discussion

We previously reported that TWIK-1-mediated currents display outwardly rectifying K^+^ currents and contributed to the intrinsic excitability of DGGCs in the mouse hippocampus^13^. In the present study, we further demonstrated that TWIK-1 in DGGCs act as a heterodimeric channel with TASK-3 and that the TWIK-1/TASK-3 heterodimeric channels contribute to the RMP and intrinsic excitability of DGGCs. Importantly, the TWIK-1/TASK-3 heterodimeric channels were inhibited by NT–NTSR1 signaling, which facilitated the excitability of the DGGCs.

The electrophysiological properties and functional roles of TWIK-1 have not been fully characterized because a detectable TWIK-1 current has not been measured in heterologous expression systems^3–5^. Recent studies have demonstrated that TWIK-1-mediated currents display outwardly rectifying K^+^ currents in cerebellar granule cells and DGGCs^5,13^. Because, TWIK-1-mediated currents were first characterized in heterologous expression systems as weakly inwardly rectifying^2,8^, TWIK-1-mediated outwardly rectifying currents were quite intriguing^5,13,25^. Interestingly, recent publications have provided evidence of heteromerization between TWIK-1 and other K2P channels, such as TASK-3 and TREK-1^5,14^. Because heteromerization between TWIK-1 and TASK-3 results in the formation of a channel with outwardly rectifying properties^5^ and TASK-3 is known to be highly expressed in mouse DGGCs^10,16,26^, we suggested that heteromerization between TWIK-1 and TASK-3 could occur in these cells, possibly explaining the outwardly rectifying properties of TWIK-1-mediated components in the whole-cell currents of DGGCs. In this study, the coexpression and association of TWIK-1 and TASK-3 in DGGCs were confirmed by single-cell RT-PCR, BiFC, immunoprecipitation, and Duolink PLA (Figs. 1 and 2). Knockdown of TWIK-1 or TASK-3 in DGGCs led to similar results (i.e., a reduction in the density of outwardly rectifying whole-cell currents) (Fig. 3). Thus, we believe that TWIK-1 is able to form a heterodimeric channel with TASK-3 in DGGCs.

A recent report of its crystal structure confirmed that TWIK-1 may assemble as a dimer via a disulfide bridge^27^. A disulfide bridge between the cysteines at residue 69 in the first extracellular loop of TWIK-1 would seem to be important for homodimer and heterodimer channels containing TWIK-1^14,26,28^. However, there is no cysteine residue in the first extracellular loop of TASK-3, and thus the TWIK-1/TASK-3 heterodimeric channel in DGGCs would not be formed via a cysteine disulfide bridge between these K2P subunits. In the present study, since BiFC signals between TWIK-1 and TASK-3 were observed in intracellular organelles, possibly the endoplasmic reticulum, Golgi apparatus, and plasma membrane (Fig. 2a), it appears that TWIK-1/TASK-3 heterodimeric channels are formed at the early step of transport of newly synthesized membrane proteins. Therefore, it is possible that there is another, yet to be identified mechanism for the dimerization of TWIK-1 and TASK-3 at the early step of membrane protein transport.

Because, the TWIK-1/TASK-3 heterodimeric channel is formed between two different subunits, it is plausible that regulatory processes known to affect both TWIK-1 and TASK-3 subunits could also affect the activities of this heterodimeric channel. Indeed, in the present study, the TWIK-1/TASK-3 heterodimeric channels in DGGCs were inhibited by NT–NTSR1 signaling, which is known to be a negative modulator of TASK-3^16^ (Fig. 6). Because the activity of TASK-3 was modulated by diverse regulating factors, including Gq-coupled G-protein-coupled receptors (GPCRs) and binding proteins, such as 14-3-3γ, βCOP, and p11^25^, it is possible that the activity of TWIK-1/TASK-3 heterodimeric channels can be modulated by these regulating factors. In particular, diverse Gq-coupled GPCRs other than NTSR1 may also be involved in mechanisms regulating TWIK-1/TASK-3 heterodimeric channels in DGGCs. In addition, TWIK-1/TASK-3 heterodimeric channels can be affected by endocytosis or SUMOylation, which regulates the activity of the TWIK-1 homodimeric channel and TWIK-1-containing heterodimeric channels^3–5,7,14,27^. Further studies are required for better understanding of the regulatory mechanisms of TWIK-1/TASK-3 heterodimeric channels.

We found that deficiency of TASK-3 or TWIK-1/TASK-3 in DGGCs resulted in predominant reductions of Cs^+^/TEA/4-AP-insensitive currents in DGGCs (Fig. 3). We also found that DGGCs deficient in TASK-3 or TWIK-1/TASK-3 exhibited small but significant depolarizing shifts in the reversal potential and RMP (Table 1). In the current clamp experiments, the relationship between the membrane voltage and the injected current in DGGCs deficient in both TWIK-1 and TASK-3 and in naive or Sc shRNA-infected DGGCs revealed a significantly smaller outwardly rectifying current density in the TWIK-1- and TASK-3-deficient cells. This observation suggests that TWIK-1/TASK-3 heterodimer-mediated currents attenuate the depolarization of DGGCs cells in response to current injection. Because the dentate gyrus is the main gateway to the hippocampus and DGGCs are able to effectively block or filter the excitatory input from the entorhinal cortex^29–31^, our results strongly suggest that TWIK-1/TASK-3 heterodimeric channels play an important role in the filtering properties of DGGCs. Thus, up- or down-regulation of TWIK-1/TASK-3 heterodimeric channel activity might modulate the intrinsic excitability of DGGCs and hence influence information processing from the entorhinal cortex to the hippocampus. Indeed, we found that inhibition of the TWIK-1/TASK-3 heterodimeric channel activity mediated by NT, which is highly expressed in the hippocampus^32–35^, increased the intrinsic excitability of DGGCs (Fig. 6).

NT is widely expressed in various regions of the brain and has been implicated in the modulation of diverse physiological and pathophysiological brain functions, such as learning, memory, schizophrenia, and Parkinson’s disease^36,37^. Thus, the link between the TWIK-1/TASK-3 heterodimeric channel and NT–NTSR1 signaling suggests that the TWIK-1/TASK-3 channel is involved in a range of brain functions and diseases in the brain. For example, the activities of the channel in cerebellar granule cells may also be modulated by the NT-mediated regulatory mechanism, given that NTSR1 and TWIK-1/TASK-3 heterodimeric channels are found in these cells^5,38^. We expect further studies will uncover the diverse functions of TWIK-1/TASK-3 heterodimeric channels via modulation of GPCRs in the brain.

In summary, the findings of this study demonstrated that TWIK-1 can form a heterodimeric channel with TASK-3 in mouse hippocampal DGGCs. The TWIK-1/TASK-3 heterodimeric channel mediates outwardly rectifying currents and determines the intrinsic excitability of DGGCs by contributing to the electrical properties of these cells. NT-mediated NTSR1 activation triggers depolarization of DGGCs by inhibiting the TWIK-1/TASK-3 heterodimeric channels, facilitating the excitability of DGGCs. Taken together, these results clearly show that TWIK-1 plays a pivotal role as a heterodimeric channel with TASK-3 in the electrical properties of DGGCs.

## Electronic supplementary material

Supplementary Figure Legends

Supplementary Figure 1

Supplementary Figure 2

Supplementary Figure 3

Supplementary Table 1
