# Supplementary material for: TWIK-1/TASK-3 heterodimeric channels contribute to the neurotensin-mediated excitation of hippocampal dentate gyrus granule cells
Source: Exp Mol Med. 2018 Nov 12;50(11):145. doi: 10.1038/s12276-018-0172-4 (PMC6230555; doi:10.1038/s12276-018-0172-4)
Supplement: Supplementary file 1 — Supplementary Figure Legends [file 12276_2018_172_MOESM1_ESM.docx]

**Supplementary Figure 1. Validation of the knockdown efficiency of TASK-3 shRNA**

**(A)** The knockdown efficiency of TASK-3 shRNA was validated by western blot using a TASK-3-specific antibody from GFP-TASK-3-expressing HEK293T cells. The graph shows the average knockdown effect of TASK-3 shRNA (n = 3). All values are means ± SEM. ** *P* < 0.01. **(B)** Representative immunostaining images using a TASK-3-specific antibody (green) in DGGCs infected with Ad- Sc shRNA or Ad-TASK-3 shRNA (red). (60×). Scale bar, 20 μm. ML: dentate molecular layer, GL: dentate granule layer, H: dentate hilus.

**Supplementary Figure 2. Effect of a high dose of potassium channel blockers in DGGCs**

**(A)** The averaged current–voltage (*I–V*) relationship of the whole-cell currents from the DGGCs infected with Ad-Sc shRNA and DGGCs from naïve animals were measured in standard artificial cerebrospinal fluid in the absence and presence of Cs^+^/TEA/4-AP (1 mM/5 mM/5 mM). **(B)** Summary bar graph showing the normalized current density, as in (A), at +50 mV. All values are means ± SEM (*** *P* < 0.001). **(C)** The averaged current–voltage (*I–V*) relationship of the whole-cell currents from COS-7 cells transfected with TASK-3 or TWIK-1/TASK-3 chimera were measured in the absence or presence of Cs^+^/TEA/4-AP (1 mM/5 mM/5 mM). **(D)** Summary bar graph showing the normalized current density, as in (C), at +40 mV. All values are means ± SEM. n.s. indicates not statistically significant.

**Supplementary Figure 3. Control experiments for NT–NTSR1 signaling-mediated TASK-3 inhibition**

**(A–D)** Averaged *I–V* relationships in COS-7 cells transfected with TASK-3 (A), NTSR1 (B), TWIK-1 (C), and a TASK-3/TWIK-1 concatenated chimera channel (D), measured before and after the application of 10 μM NT in a whole-cell configuration. **(E)** Summary bar graph showing the normalized current density, as in (A–D), at +40 mV. **(F)** Bar graph showing the inhibition ratio with NT application. All values are means ± SEM.
