# Supplementary figures and images for: TWIK-1/TASK-3 heterodimeric channels contribute to the neurotensin-mediated excitation of hippocampal dentate gyrus granule cells
Source: Exp Mol Med. 2018 Nov 12;50(11):145. doi: 10.1038/s12276-018-0172-4 (PMC6230555; doi:10.1038/s12276-018-0172-4)

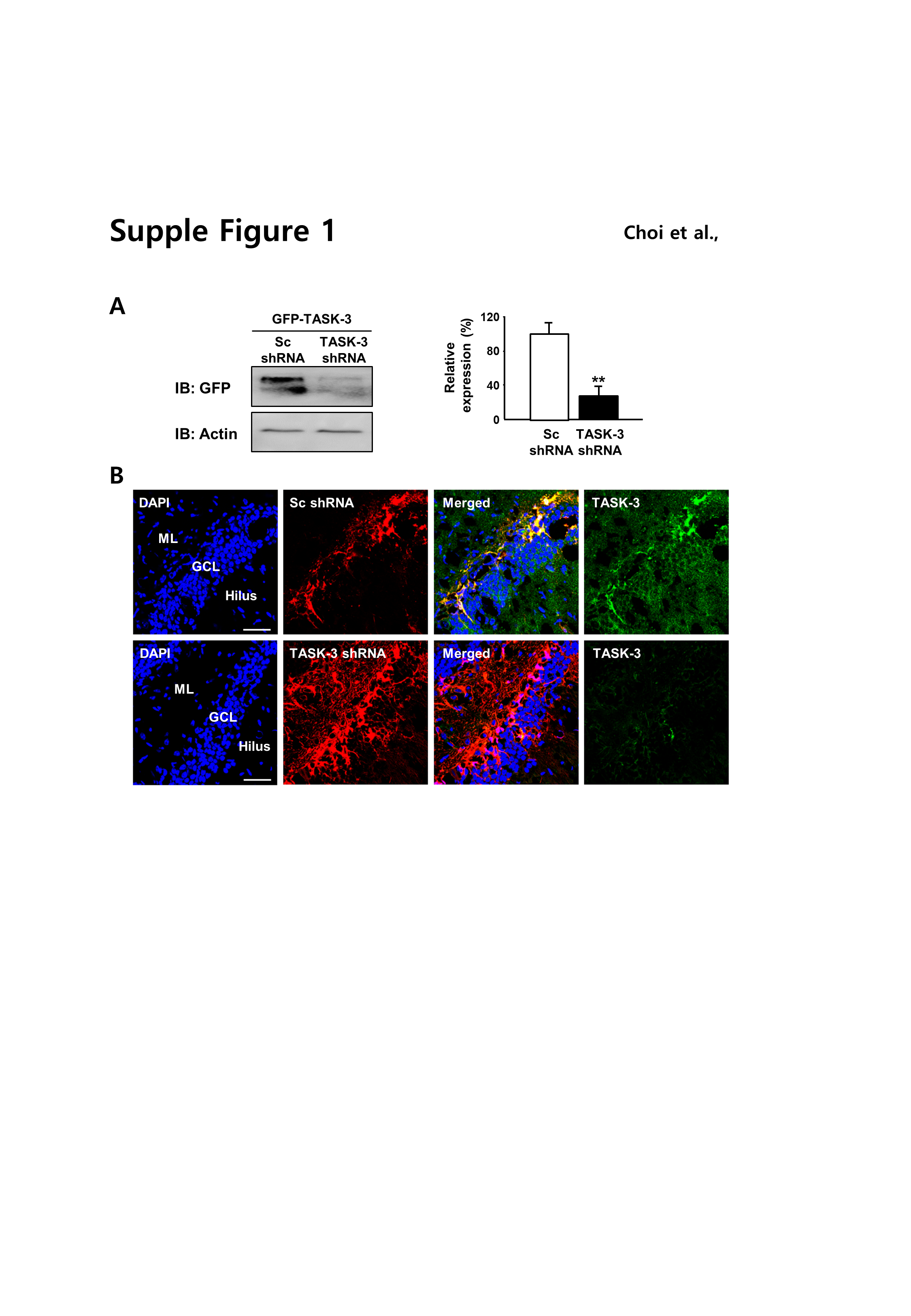

Supplement: Supplementary file 2 — Supplementary Figure 1 [file 12276_2018_172_MOESM2_ESM.tif]

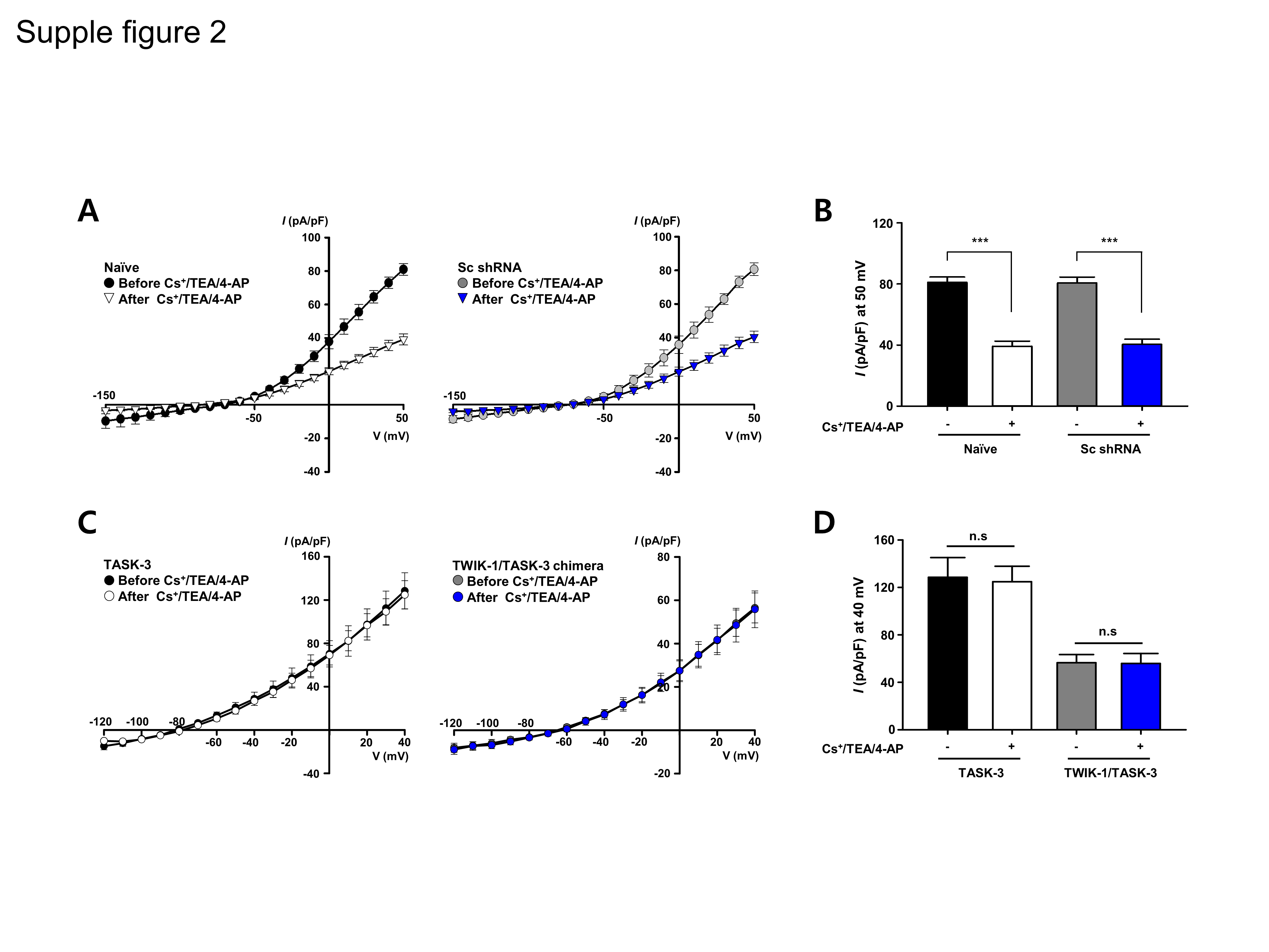

Supplement: Supplementary file 3 — Supplementary Figure 2 [file 12276_2018_172_MOESM3_ESM.tif]

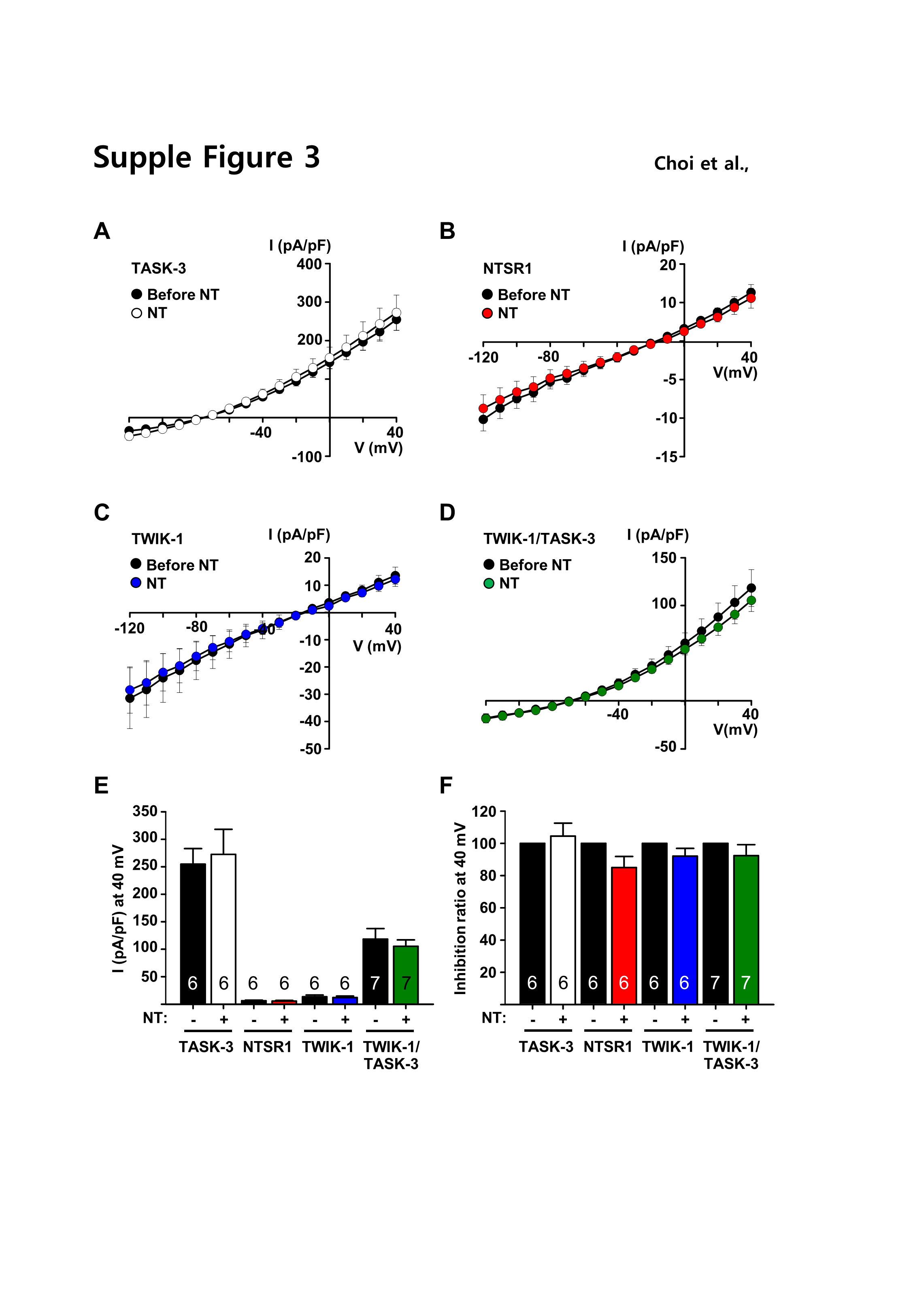

Supplement: Supplementary file 4 — Supplementary Figure 3 [file 12276_2018_172_MOESM4_ESM.tif]

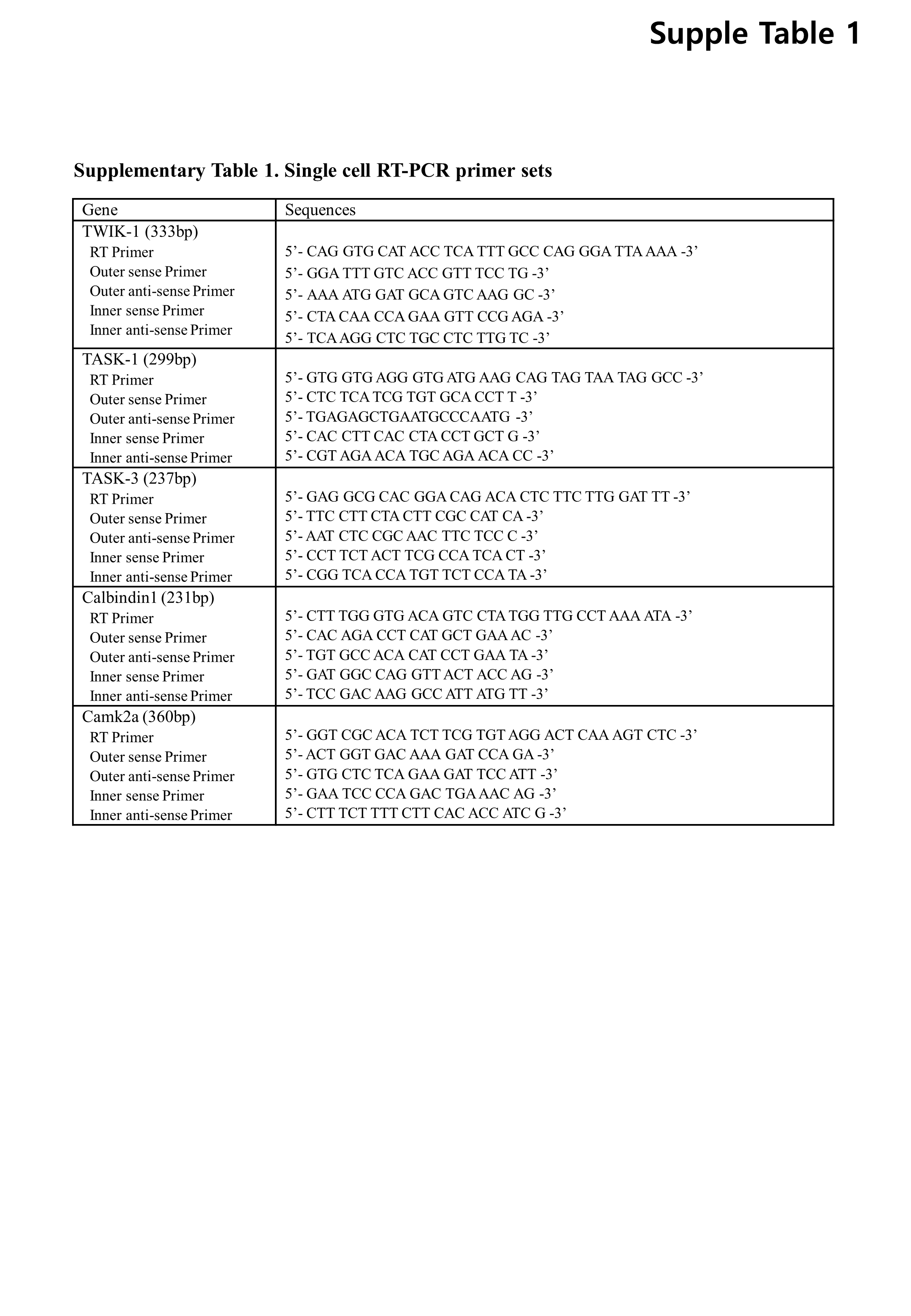

Supplement: Supplementary file 5 — Supplementary Table 1 [file 12276_2018_172_MOESM5_ESM.tif]
